# Supplementary material for: Finely Tunable Carbon Nanofiber Catalysts for the Efficient Production of HMF in Biphasic MIBK/H2O Systems
Source: Nanomaterials (Basel). 2024 Jul 31;14(15):1293. doi: 10.3390/nano14151293 (PMC11314515; doi:10.3390/nano14151293)
Supplement: Supplementary file 1 [file nanomaterials-14-01293-s001.zip › nanomaterials-3028465-supplementary.pdf]

Supporting information

Finely tunable CNFs catalysts for the efficient production of HMF in biphasic MIBK/H_2O systems

Charf Eddine Bounoukta [a,b], Cristina Megías-Sayago [,c], Nuria Rendón [c], Fatima Ammari [b], Miguel Angel Centeno [a] and Svetlana Ivanova [a*]

[a]Departamento de Química Inorgánica e Instituto de Ciencia de Materiales de Sevilla, Centro Mixto CSIC-Universidad de Sevilla, 41092 Sevilla, Spain.
[b]Laboratoire de Génie des Procédés Chimiques- LGPC, Département de Génie des Procéés, Faculté de Technologie, Université Ferhat Abbas Setif-1, 19000 Setif, Argelia.
[c]Departamento de Química Inorgánica e Instituto de Investigaciones Químicas, Centro mixto CSIC-Universidad de Sevilla, 41092 Sevilla, Spain
* Correspondence: author: sivanova@us.es, svetlana@icmse.csic.es

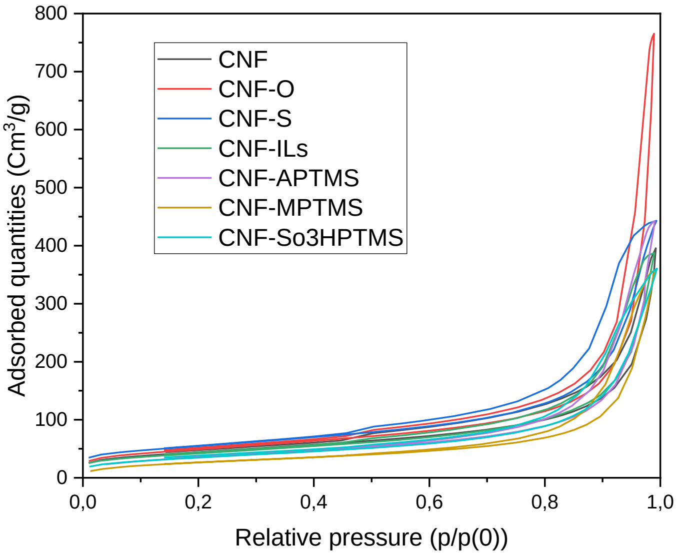

Figure SI1. *N_2 physisorption isotherms of the CNF based catalysts.*

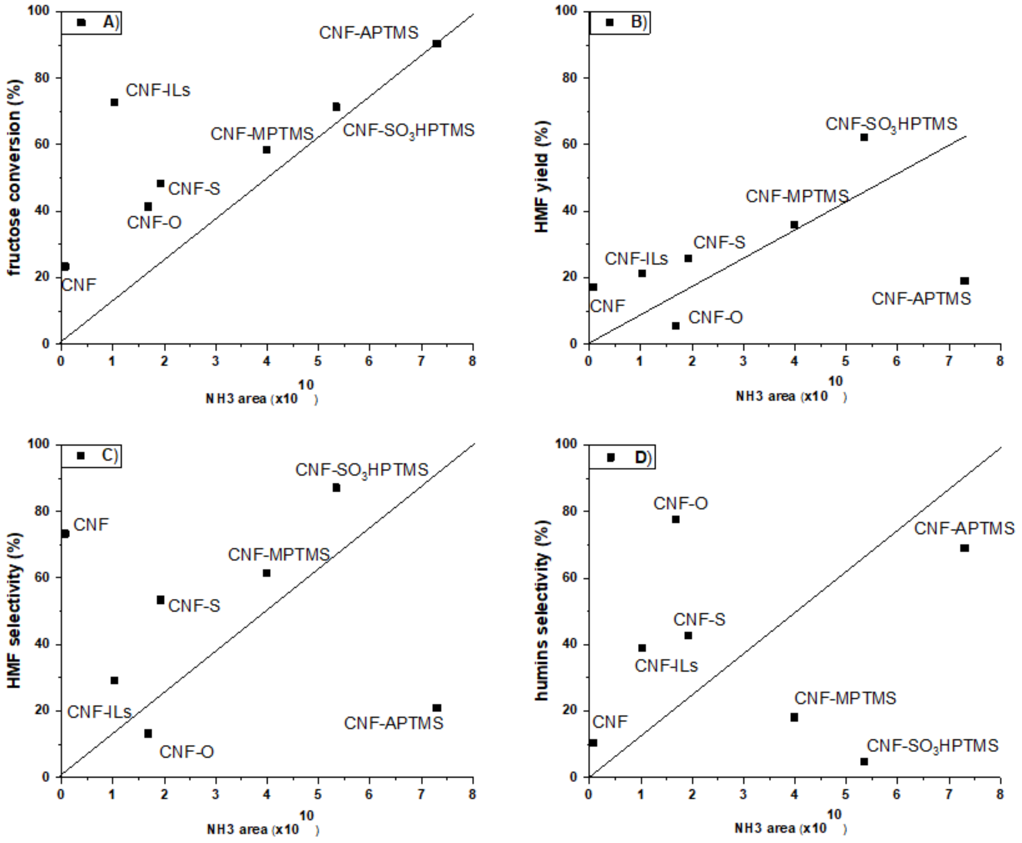

Figure SI2. Calculated rection parameters vs NH_3 area plot. A) fructose conversion, B) HMF yield, C) HMF selectivity, D) humins selectivity).

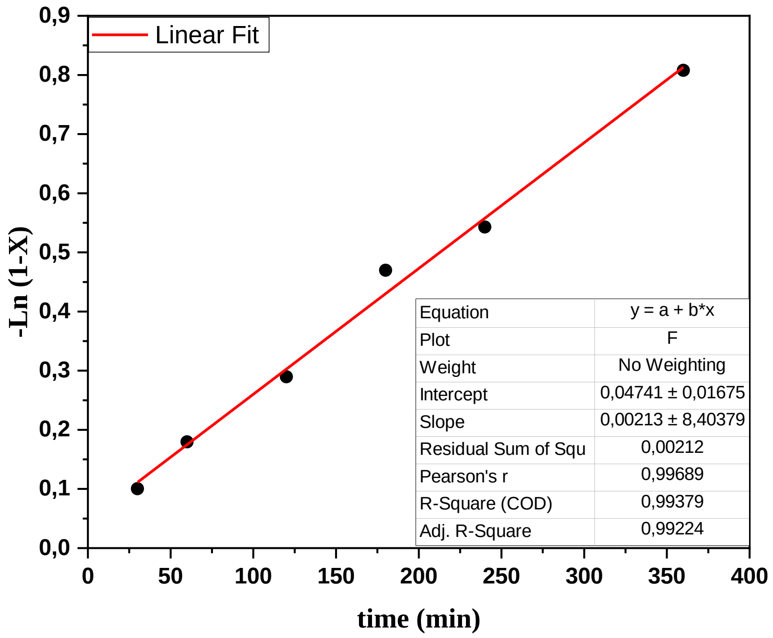

Figure SI3. *The kinetics profile of fructose dehydration (fitted by 1st order assumption) over* SO_3*HPTMS catalyst.*

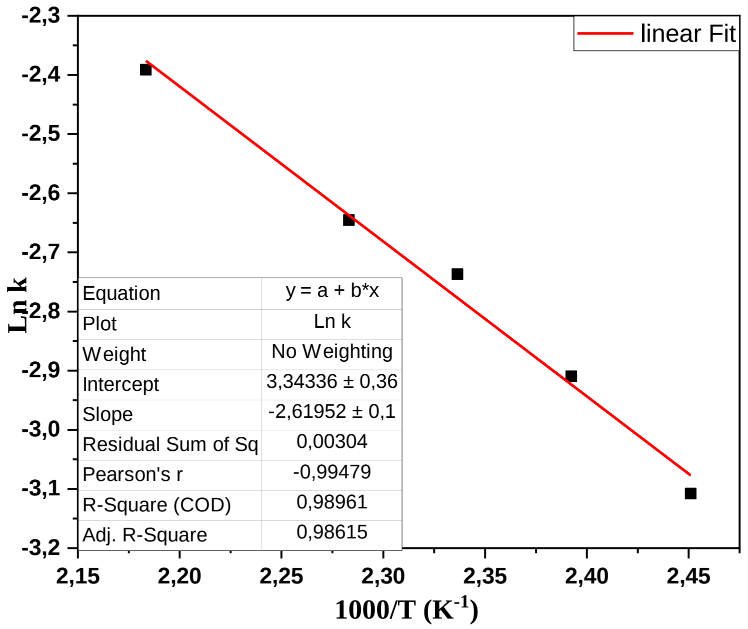

Figure SI4. *Arrhenius plot for first order fructose dehydration reaction over CNF-SO_3HPTMS catalyst.*

Table SI1. kinetic parameters for the global reaction of dehydration of fructose and Ea for fructose dehydration over SO_3H-PTMS-CNF catalyst

**Temperature** (°C)	135	145	155	165	185
**K 10^3** (min^{-1})	0.78	1.23	1.83	2.13	4.06
**Ea** (Kj mol^{-1})	21.78				
